# Fibrinogen in Alzheimer’s Disease, Parkinson’s Disease and Lewy Body Dementia: A Mendelian Randomization Study

**DOI:** 10.3389/fnagi.2022.847583

**Published:** 2022-07-06

**Authors:** Hanyu Zhang, Zengyuan Zhou

**Affiliations:** ^1^Department of General Practice, Clinical Medical College and Affiliated Hospital of Chengdu University, Chengdu, China; ^2^Department of Nutrition, Chengdu Women’s and Children’s Central Hospital, School of Medicine, University of Electronic Science and Technology of China, Chengdu, China

**Keywords:** fibrinogen, Alzheimer’s disease, Parkinson’s disease, Lewy body dementia, Mendelian randomization

## Abstract

Fibrinogen is reportedly associated with neurodegenerative diseases (NDs), but the underlying causality remains controversial. Using Mendelian randomization (MR), this study aimed to assess the causal association between fibrinogen and Alzheimer’s disease (AD), Parkinson’s disease (PD), and Lewy body dementia (LBD). Genetic variants associated with fibrinogen and γ-fibrinogen were selected and used as instrumental variables. The effect estimates of the main analysis were obtained by inverse-variance weighting (IVW), complemented by sensitivity analyses to verify model assumptions, and multivariable MR was conducted to control for potential pleiotropic effect. Two-step MR was performed to assess the causal association through mediators. The main analysis suggested no causal association between genetically predicted plasma fibrinogen and γ-fibrinogen levels and the risk of AD, PD, and LBD. The effect estimates did not change in the follow-up sensitivity analyses and MVMR. However, the two-step MR analysis provides evidence that fibrinogen may contribute to the risk of AD via CRP levels. There was an inverse effect of adult height levels on the risk of AD. Our results support the effects of fibrinogen on the risk of AD through increasing plasma CRP levels. Our study found no evidence to support the effects of genetically determined fibrinogen and γ-fibrinogen levels on the risk of PD and LBD. Additionally, our findings suggested an inverse association between genetically determined adult height levels and the risk of AD. Future studies are needed to elucidate the underlying mechanisms and their clinical applications.

## Introduction

Due to an aging population, neurodegenerative diseases (NDs) are one of the most serious public health burdens and causes of mortality and morbidity worldwide (GBD 2016 Neurology Collaborators, 2017). So far, there are few or no effective curative treatment options for NDs, although not for lack of effort. The processes of NDs begin long before clinical symptoms appear, but the lack of effective biomarkers prevents patients from being diagnosed in the early stages of these diseases. Hence, there is an urgent need to identify the risk factors that cause NDs and appraise patients at risk in order to allow for prevention and early intervention to slow down the course of these disorders. For many years, vascular factors and inflammatory responses were considered to be involved in the pathological process of some NDs, such as Alzheimer’s disease (AD), Parkinson’s disease (PD), and Lewy body dementia (LBD) (Vemuri et al., 2015; Voet et al., 2019). Fibrinogen is a pleiotropic protein of the hemostatic system and inflammatory response (Sidelmann et al., 2000; Adams et al., 2007). The component is made up of three polypeptide chains named Aα, Bβ, and γ, which the γ chain accounting for around 8∼15% of total circulating fibrinogen (Cheung et al., 2009). Structurally, fibrinogen comprises receptors expressed by nervous system cells and binding sites for regulatory proteins of pivotal nervous system functions (Petersen et al., 2018). Recent studies have shown that fibrinogen plays a major role in NDs due to its involvement in the coagulation cascade (Sidelmann et al., 2000) and inflammatory system (Davalos and Akassoglou, 2012). As such, a growing number of studies have attempted to explore the potential role of fibrinogen in AD, PD and LBD (Chiam et al., 2015; Petersen et al., 2018; Zhou et al., 2020). However, the results of conventional observational studies may have been affected by residual confounding and reverse causation (Phillips and Smith, 1991), thus limiting our knowledge of the effect fibrinogen on the risk of NDs.

**FIGURE 1:**
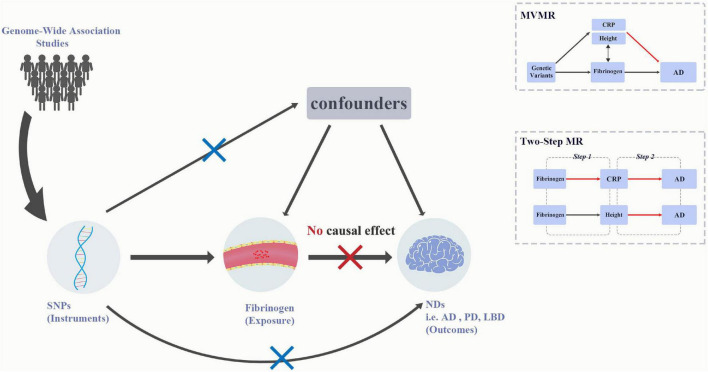
Graphical abstract for current univariable MR and MVMR analyses. MR analysis is based on the three assumptions: (1) IVs should be associated with the exposure, (2) any association between the IVs and the outcome must come via the IVs association of IVs with the exposure, and (3) IVs should be independent of any measured and unmeasured confounders. The SNPs used in MR acted as IVs were obtained from large-scale genome-wide association studies. Results for univariable MR did not support the causal effect of fibrinogen and γ-fibrinogen on AD, PD, and LBD, whereas the results for MVMR showed the association between height and AD. AD, Alzheimer’s disease; IVs, instrumental variables; LBD, dementia with Lewy bodies; MR, Mendelian randomization; MVMR, multivariable Mendelian randomization; NDs, Neurodegenerative diseases; PD, Parkinson’s Disease.

Mendelian randomization (MR) is a method that uses genetic variants, or single-nucleotide polymorphisms (SNPs), as instruments to infer causal effect of lifelong risk factors (exposure) on diseases (outcome). This method can reduce the impact of confounding and reverse causation (Smith and Ebrahim, 2003). In the current study, we performed the univariable and multivariable MR analyses to assess the associations between the genetically determined circulating fibrinogen and γ-fibrinogen levels and the risk of AD, PD and LBD (Figure 1). In addition, we extend our study using a two-step MR analysis to assess the causal effects of fibrinogen and γ-fibrinogen levels on AD through the potential mediators.

## Materials and Methods

### Data Sources

#### Genetic Instruments Associations With Fibrinogen

We obtained the SNPs with fibrinogen-related phenotypes from the published genome-wide association study (GWAS) summary meta-analysis statistics (Sabater-Lleal et al., 2013). This study was comprised of 28 GWAS, including more than 90,000 individuals of European ancestry. Then, the SNPs with γ-fibrinogen levels were obtained from a recent GWAS of European ancestry from the Atherosclerosis Risk in Communities (ARIC) study (Maners et al., 2020). To validate and select the SNPs for plasma fibrinogen levels, we chose genetic instruments that were genome-wide significant (5*10^–8^). Additionally, the F statistics of each SNPs of at least 10 is considered as sufficient for performing the MR analysis, the calculation formula is as following: F = *R*^2*^ (N–2)/(1–*R*^2^).

#### Outcome Datasets

Genetic variants associated with AD were obtained from the published genome-wide meta-analysis (Jansen et al., 2019), which includes data consisting of 71,880 cases of clinically diagnosed AD and AD-by-proxy, based on parental diagnoses, and 383,378 controls of European ancestry from four consortia: the Alzheimer’s-disease working group of the Psychiatric Genomics Consortium (PGC-ALZ), the International Genomics of Alzheimer’s Project (IGAP), the Alzheimer’s Disease Sequencing Project (ADSP) and the UK Biobank. Summary statistics of PD risk for the selected instrument variables, which included 33,674 PD cases and 449,056 controls of European ancestry, were acquired from the published meta-analysis of PD GWAS from International Parkinson’s Disease Genomics Consortium (IPDGC) (Nalls et al., 2019). Summary statistics with LBD were obtained from a recent published GWAS (Chia et al., 2021). That study recruited 2,591 individuals diagnosed with LBD and 4,027 neurologically healthy individuals across 44 institutions or consortia.

Details of the participant data for each dataset are described in the original publication.

### Statistical Analysis

#### Mendelian Randomization

In the current study, we used several MR methods to estimate the causal associations between plasma fibrinogen levels and AD, PD, and LBD. The inverse variance-weighted (IVW) estimates were used for principal MR analysis, and the fixed-effects (FE) model was applied to homogeneous data, while the multiplicative random (RE) model was suitable for heterogeneous data (Burgess et al., 2019). Results are presented as odds ratios (OR) for each outcome risk per corresponding unit change in fibrinogen levels on natural logarithm transformation.

#### Sensitivity Analyses

For sensitivity analyses, we performed MR-Egger regression analysis (Bowden et al., 2015), maximum likelihood estimate (Pierce and Burgess, 2013), weighted-median estimator (Bowden et al., 2016) and MR Pleiotropy Residual Sum and Outlier (MR-PRESSO) methods (Verbanck et al., 2018) to verify the main MR assumptions. The MR-Egger regression, which requires the InSIDE (instrument strength independent of direct effects) assumption to be valid, performs a weighted linear regression of SNP outcome on the SNP fibrinogen effect estimates, which provides a valid effect estimate even when all the SNPs are invalid instruments. Then, the MR-Egger intercept estimates the overall unbalanced horizontal pleiotropy effect across the genetic variants. For the maximum likelihood estimate method, estimates of the probability distribution parameters can be obtained by maximizing of the likelihood. A weighted-median method can provide a consistent effect estimate if at least 50% of the genetic variants are valid. We then used the MR-PRESSO analysis to detect horizontal pleiotropy and outlier SNPs. Cochran’s Q statistic for the IVW method was applied to test the heterogeneity of the genetic variants (Greco et al., 2015). The Radial MR method was performed to detect heterogeneity and outliers (Bowden et al., 2018). Additionally, the leave-1-out analysis were performed to determine whether the results were disproportionately affected by any single variants. The power calculations were estimated for the primary analyses using online tool (Brion et al., 2013; Supplementary Table 5). Finally, we conducted Bonferroni correction for multiple comparisons of 4 exposures and 3 outcomes (*P*-value threshold of 0.05/7 = 0.0071). Results that were significant before correction for multiple comparisons but not after correction were considered suggestive.

### Multivariable Mendelian Randomization

To correct the effect of the potential confounders on fibrinogen levels, we performed the Multivariable MR (MVMR) analysis (Sanderson, 2021) based on the existing biological knowledge and potential pleiotropy on IVs of the exposure. Given the role of fibrinogen in inflammation (Davalos and Akassoglou, 2012), we performed the MVMR analysis adjusting the fibrinogen levels for CRP levels. Additionally, using the online database “PhenoScanner” (Staley et al., 2016) to detect potentially related phenotypes, we found that SNPs for fibrinogen (rs2706383) and γ-fibrinogen (rs59950280) levels are associated with height. Thus, we also performed MVMR analyses adjusting fibrinogen and γ-fibrinogen levels for height to adjust for potential genetic pleiotropy.

The MVMR analysis used summary level data from three GWAS. We used a GWAS of fibrinogen and γ-fibrinogen levels from population-based study of 10,708 European individuals (Pietzner et al., 2020). Genetic variants of CRP levels were obtained from a published GWAS involving 3,301 individuals of European descent (Sun et al., 2018). Then, we used GWAS summary data of adult height from a published study from the Genetic Investigation of ANthropometric Traits (GIANT) consortium (Berndt et al., 2013).

### Two-Step Mendelian Randomization

Finally, we used a two-step MR analysis to assess the association of the exposure with the outcome, exposure with mediators, and mediators with the outcome. In the first step, we estimated the causal effect of fibrinogen and γ-fibrinogen levels on CRP levels and adult height. In the second step, we estimated the causal effect of CRP levels and adult height on the risk of AD.

All statistical analyses were performed with the “TwoSampleMR” and “MRPRESSO” and “MendelianRandomization” packages (Yavorska and Burgess, 2017; Hemani et al., 2018; Verbanck et al., 2018) in R, version 4.1.2.^1^

### Data Availability

The datasets analyzed in this study are publicly available summary statistics. Summary statistics for PD, CRP, and height were obtained from MRC Integrative Epidemiology Unit (IEU) OpenGWAS database (Elsworth et al., 2020). Summary level data for fibrinogen, γ-fibrinogen, AD and LBD were downloaded from the NHGRI-EBI GWAS Catalog (Buniello et al., 2019).

## Results

### Selection of Genetic Instruments

We obtained the 23 SNPs associated with plasma fibrinogen levels at a genome-wide significance level. One SNP (rs16844401) was excluded because of F-statistics lower than 10. One of the instruments (rs1019670) was removed due to linkage disequilibrium clumping (*r*^2^< 0.1). After harmonizing the instruments and each outcome dataset, 18, 17, and 18 remaining SNPs were used as genetic instruments for causal estimation between fibrinogen levels with AD, PD, and LBD. Additionally, 16 SNPs were selected as genetic instruments for γ-fibrinogen. After harmonizing the instruments and the AD, PD, and LBD datasets, 14, 15, 14 SNPs remained for causal estimation between γ-fibrinogen and each outcome. Detailed information on the instruments associated with fibrinogen and each outcome is displayed in Supplementary Tables 1A,B.

### Mendelian Randomization Analysis

**FIGURE 2:**
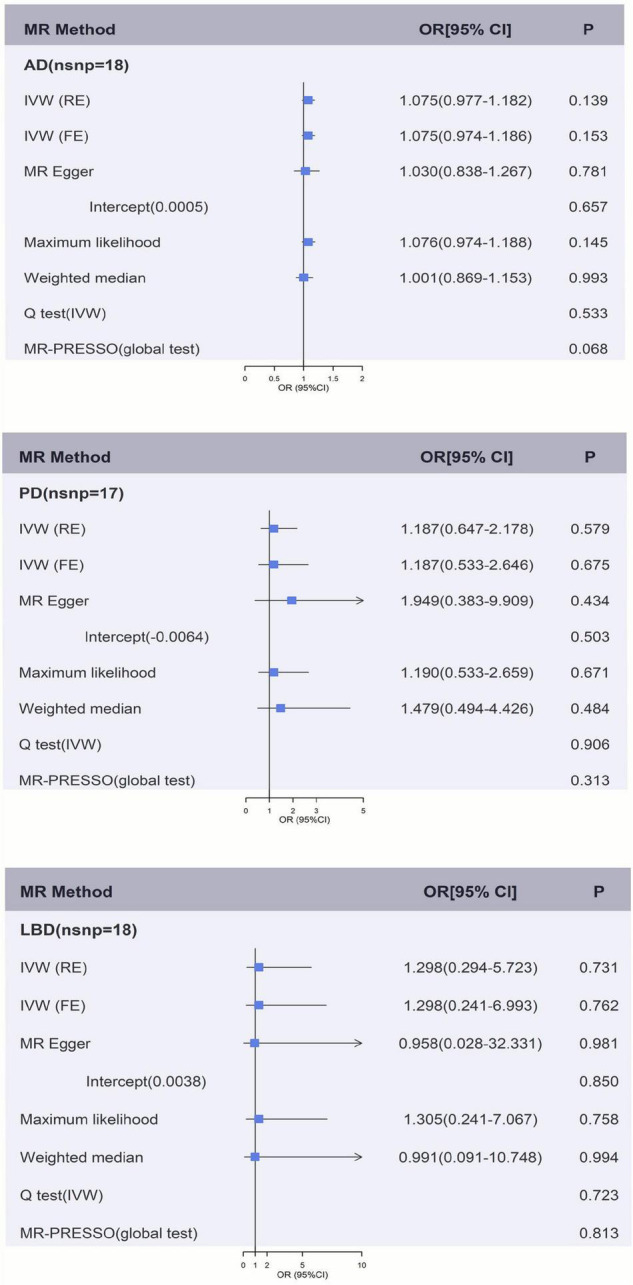
Genetically predicted fibrinogen levels with risk of AD, PD, and LBD. Results of the primary MR and sensitivity analyses. Estimated ORs per unit increase in log-transformed fibrinogen for each outcome. AD, Alzheimer’s disease; IVW, Inverse variance weighting; LBD, Lewy body dementia; FE, fixed effects; MR, Mendelian randomization; OR, odds ratio; PD, Parkinson’s Disease; RE, random effects.

**FIGURE 3:**
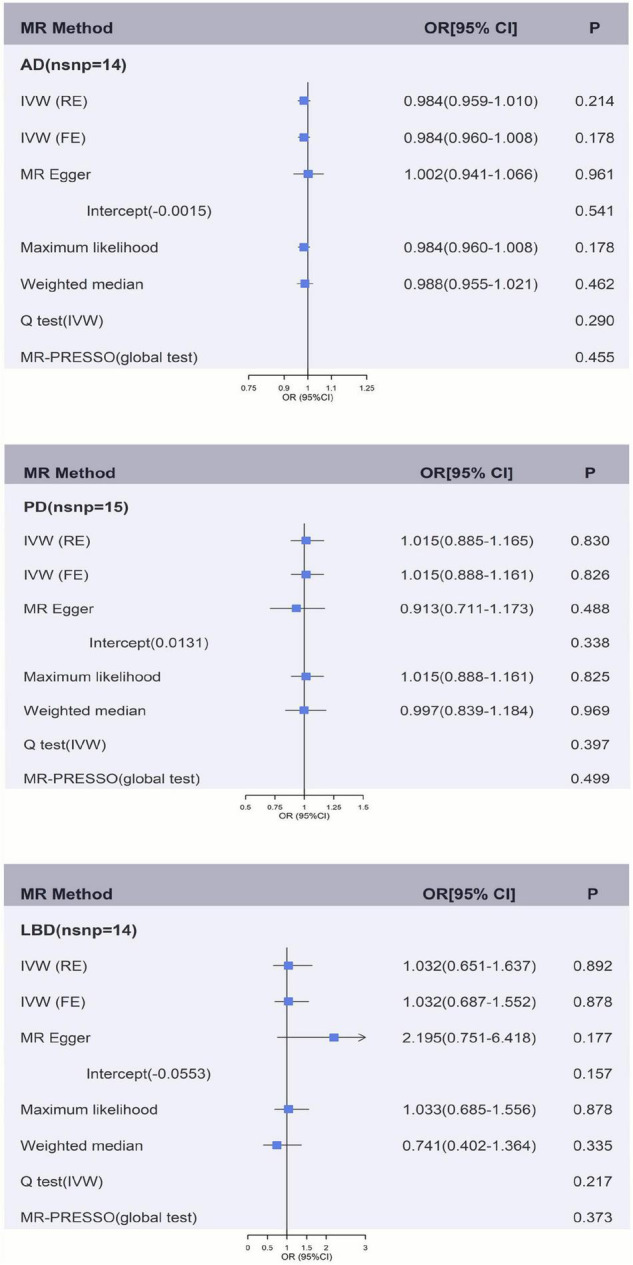
Genetically predicted γ-fibrinogen levels with risk of AD, PD, and LBD. Results of the primary MR and sensitivity analyses. Estimated ORs per unit increase in log-transformed γ-fibrinogen for each outcome. AD, Alzheimer’s disease; IVW, Inverse variance weighting; LBD, Lewy body dementia; FE, fixed effects; MR, Mendelian randomization; OR, odds ratio; PD, Parkinson’s Disease; RE, random effects.

Figures 2, 3 and Supplementary Tables 2A,B showed the causal effect on genetically elevated fibrinogen and γ-fibrinogen levels with each outcome. In the MR analysis using IVW, we found that genetically determined fibrinogen levels were not associated with AD [random effects OR = 1.075 (95%CI:0.977, 1.182), *P* = 0.139], PD [random effects OR = 1.187 (95%CI:0.647, 2.178), *P* = 0.579] and LBD [random effects OR = 1.298 (95%CI:0.294, 5.723), *P* = 0.731]. The same results were observed in the MR-Egger regression, weighted median method and maximum likelihood approach. Genetically determined γ-fibrinogen levels were not associated with AD [random effects OR = 0.984 (95%CI:0.959, 1.010), *P* = 0.214], PD [random effects OR = 1.015 (95%CI:0.885, 1.165), *P* = 0.830], and LBD [random effects OR = 1.032 (95%CI:0.651, 1.637), *P* = 0.892] using IVW analysis. The results were consistent across the MR-Egger regression, weighted median method and maximum likelihood approach.

### Sensitivity Analysis

Figures 2, 3 and Supplementary Tables 2A, 3B show the sensitivity analysis for each outcome in this study. The *P*-values from the MR-Egger intercept test were not statistically significant therefore a strong pleiotropic bias was not found. The results of the Cochran’s *Q*-test showed no apparent heterogeneity between individual SNP estimations. In addition, the analysis of MR-PRESSO test did not identify any outliers for any of the estimates. In the leave-one-out analysis, we did not find the risk estimates of genetically predicted fibrinogen levels on PD and LBD changed after excluding one SNP iteratively, indicating that there were no potentially influential SNPs could affect the causal association. However, the result of IVW-based leave-one-out analysis showed a significant association between fibrinogen and AD after the exclusion of rs4129267 (*P* = 0.041), but this association is not robust and stable.

### Multivariable Mendelian Randomization

Using summary level data for each phenotype, univariable MR provided significant evidence that genetically determined fibrinogen [fixed effects OR = 1.030 (95%CI:1.003, 1.058), *P* = 0.003] and γ-fibrinogen [fixed effects OR = 1.027 (95%CI:1.001, 1.055), *P* = 0.042] levels were associated with AD. The results of univariable MR showed that fibrinogen and γ-fibrinogen were not associated with PD [IVW_*fib*_ fixed effects OR = 1.063 (95%CI:0.866, 1.305), *P* = 0.560] [IVW_γ_ fixed effects OR = 0.982 (95%CI:0.809, 1.190), *P* = 0.850] and LBD [IVW_*fib*_ fixed effects OR = 0.912 (95%CI:0.581, 1.431), *P* = 0.688] [IVW_γ_ fixed effects OR = 0.837 (95%CI:0.558, 1.256), *P* = 0.053]. In the results of the fixed effects IVW-based MVMR, the effect of fibrinogen on AD did not change substantially after adjustment for height [fixed effects OR = 1.024 (95%CI:1.0005, 1.049), *P* = 0.043], whereas the results of random effects IVW showed a null effect [random effects OR = 1.024 (95%CI:0.999, 1.051), *P* = 0.065]. It suggests the results are not robust and stable. Results of MVMR showed that genetically determined fibrinogen levels were not associated with PD [random effects OR = 1.063 (95%CI:0.857, 1.319), *P* = 0.581] and LBD [random effects OR = 0.864 (95%CI:0.557, 1.340), *P* = 0.513] after adjustment for height. Then, after adjustment for height, genetically predicted γ-fibrinogen levels showed no association with AD [random effects OR = 1.018 (95%CI:0.993, 1.044), *P* = 0.145], PD [random effects OR = 0.992 (95%CI:0.797, 1.236), *P* = 0.941] and LBD [random effects OR = 0.821 (95%CI:0.535, 1.261), *P* = 0.368]. However, the results of MVMR indicated that adult height levels were inversely associated with the risk of AD [random effects OR = 0.996 (95%CI:0.992, 0.99992), *P* = 0.023]. In the results of IVW-based MVMR, there was no evidence of indirect effects through fibrinogen levels for AD, PD, and LBD after adjustment for CRP. Similarly, we did not observe a significant effect of γ-fibrinogen on each outcome after adjusting for CRP. The MVMR results are presented in Table 1 and Supplementary Tables 4A,B.

**TABLE 1:** Results of multivariable MR adjusting for the effect of height and CRP.

Outcomes	Exposures						
		IVW (RE)		IVW (FE)		MR Egger	
		OR (95% CI)	P	OR (95% CI)	P	OR (95% CI)	*P*
**AD**							
	Fibrinogen	1.024 (0.999–1.051)	0.065	1.024 (1.000–1.049)	0.043*	1.019 (0.990–1.050)	0.219
	Height	0.996 (0.992–0.9999)	0.022*	0.996 (0.992–0.9999)	0.012*	0.995 (0.991–0.999)	0.02*
	Fibrinogen	1.025 (0.990–1.062)	0.166	1.025 (0.998–1.054)	0.072	0.976 (0.919–1.374)	0.438
	CRP	1.013 (0.993–1.033)	0.197	1.013 (0.997–1.029)	0.094	0.964 (0.910–1.020)	0.197
	γ-fibrinogen	1.018 (0.993–1.044)	0.145	1.018 (0.996–1.040)	0.11	1.014 (0.985–1.044)	0.344
	Height	0.996 (0.992–0.9999)	0.023*	0.996 (0.992–0.9999)	0.012*	0.995 (0.991–0.999)	0.021*
	γ-fibrinogen	1.023 (0.994–1.054)	0.134	1.023 (0.996–1.052)	0.096	0.991 (0.940–1.045)	0.734
	CRP	1.012 (0.996–1.028)	0.139	1.012 (0.996–1.028)	0.099	0.985 (0.945–1.027)	0.475
**PD**							
	Fibrinogen	1.063 (0.857–1.319)	0.581	1.063 (0.888–1.273)	0.508	1.068 (0.828–1.378)	0.611
	Height	1.004 (0.969–1.040)	0.81	1.004 (0.975–1.034)	0.773	1.004 (0.969–1.040)	0.807
	Fibrinogen	1.052 (0.852–1.300)	0.635	1.052 (0.852–1.300)	0.635	1.047 (0.659–1.663)	0.847
	CRP	1.035 (0.922–1.161)	0.568	1.035 (0.922–1.161)	0.568	1.028 (0.663–1.595)	0.901
	γ-Fibrinogen	0.992 (0.797–1.236)	0.941	0.992 (0.828–1.188)	0.928	1.039 (0.799–1.351)	0.773
	Height	1.005 (0.970–1.041)	0.775	1.005 (0.976–1.035)	0.727	1.006 (0.969–1.044)	0.741
	γ-Fibrinogen	0.972 (0.798–1.185)	0.781	0.972 (0.798–1.185)	0.781	1.147 (0.784–1.677)	0.48
	CRP	1.043 (0.929–1.171)	0.474	1.043 (0.929–1.171)	0.474	1.184 (0.898–1.561)	0.228
**LBD**							
	Fibrinogen	0.864 (0.557–1.340)	0.513	0.864 (0.577–1.294)	0.479	0.899 (0.542–1.491)	0.68
	Height	1.031 (0.963–1.105)	0.376	1.031 (0.967–1.100)	0.337	1.034 (0.963–1.109)	0.358
	Fibrinogen	0.868 (0.547–1.379)	0.552	0.868 (0.547–1.379)	0.552	0.847 (0.306–2.343)	0.75
	CRP	1.133 (0.877–1.465)	0.339	1.133 (0.877–1.465)	0.339	1.104 (0.418–2.913)	0.841
	γ-Fibrinogen	0.821 (0.535–1.261)	0.368	0.821 (0.553–1.220)	0.33	0.791 (0.478–1.310)	0.362
	Height	1.031 (0.963–1.105)	0.375	1.031 (0.969–1.098)	0.337	1.029 (0.959–1.105)	0.423
	γ-Fibrinogen	0.803 (0.530–1.216)	0.3	0.803 (0.530–1.216)	0.3	0.931 (0.412–2.103)	0.862
	CRP	1.149 (0.891–1.483)	0.286	1.149 (0.891–1.483)	0.286	1.276 (0.729–2.236)	0.392

AD, Alzheimer’s disease; CRP, C-reactive protein; DLB, dementia with Lewy bodies; MR, Mendelian randomization; OR, odds ratio; PD, Parkinson’s disease. *p < 0.05.

### Two-Step Mendelian Randomization

In the first step, genetically predicted fibrinogen levels were associated with the CRP levels [random effects OR = 5.952 (95%CI:2.212, 16.015), *P* = 4.12E-04]. However, there was no association between genetically predicated fibrinogen levels and taller adult height [random effects OR = 0.736 (95%CI:0.202, 2.687), *P* = 0.643]. In the second step, we found a suggestive association between genetically predicted CRP levels and the risk of AD [random effects OR = 1.015 (95%CI:1.002, 1.028), *P* = 0.028]. We also found a suggestive association between genetically predicted taller adult height and the risk of AD [random effects OR = 0.996 (95%CI:0.992, 0.999), *P* = 0.024]. The results of random effects IVW showed a causal effect of genetically predicted γ-fibrinogen levels on taller adult height [random effects OR = 1.693 (95%CI:1.346, 2.129), *P* = 6.78E-06]. However, the sensitivity analyses were inconsistent. There was no causal effect of genetically predicted γ-fibrinogen levels on CRP levels [random effects OR = 1.181 (95%CI:0.972, 1.436), *P* = 0.094]. The two-step MR results are presented in Table 2.

**TABLE 2:** Results of two-step MR analysis.

	Exposure	Outcome	MR Method	OR[95% CI]	*P*
***Step 1***					
	**Fibrinogen**	**CRP**			
			IVW (RE)	5.952 (2.212, 16.015)	4.12E-04*
			IVW (FE)	5.952 (1.897, 18.678)	2.23E-03*
			MR Egger	2.530 (0.235, 27.239)	0.455
			Intercept		0.433
			Weighted median	4.942 (0.947, 25.786)	0.058
			Maximum likelihood	6.233 (1.974, 19.678)	1.81E-03*
			*Q*-test		0.754
			MR-PRESSO (global test)		0.784
	**Fibrinogen**	**Height**			
			IVW (RE)	0.736 (0.202, 2.687)	0.643
			IVW (FE)	0.736 (0.196, 2.760)	0.650
			MR Egger	5.332 (0.343, 82.771)	0.250
			Intercept		0.127
			Weighted median	1.256 (0.182, 8.674)	0.817
			Maximum likelihood	0.741 (0.196, 2.793)	0.658
			*Q*-test		0.498
			MR-PRESSO (global test)		0.49
	**γ-Fibrinogen**	**CRP**			
			IVW (RE)	1.181 (0.972, 1.436)	0.094
			IVW (FE)	1.181 (0.986, 1.416)	0.071
			MR Egger	1.081 (0.740, 1.581)	0.694
			Intercept		0.599
			Weighted median	1.190 (0.938, 1.509)	0.151
			Maximum likelihood	1.185 (0.989, 1.420)	0.066
			*Q*-test		0.297
			MR-PRESSO (global test)		0.419
	**γ-Fibrinogen**	**Height**			
			IVW (RE)	1.692 (1.346, 2.129)	6.78E-06*
			IVW (FE)	1.692 (0.839, 3.414)	0.141
			MR Egger	0.683 (0.007, 63.199)	0.884
			Intercept		0.729
			Weighted median	1.692 (0.776, 3.687)	0.186
			Maximum likelihood	1.693 (0.837, 3.424)	0.143
			*Q*-test		0.956
			MR-PRESSO (global test)		0.960
***Step 2***					
	**CRP**	**AD**			
			IVW (RE)	1.015 (1.002, 1.028)	0.028*
			IVW (FE)	1.015 (1.0003, 1.030)	0.046*
			MR Egger	1.062 (0.767, 1.469)	0.780
			Intercept		0.831
			Weighted median	1.010 (0.992, 1.028)	0.281
			Maximum likelihood	1.015 (1.0001, 1.030)	0.048*
			Q test		0.439
	**Height**	**AD**			
			IVW (RE)	0.996 (0.992, 0.999)	0.024*
			IVW (FE)	0.996 (0.992, 0.999)	0.016*
			MR Egger	0.977 (0.961, 0.995)	0.014*
			Intercept		0.039*
			Weighted median	0.995 (0.990, 1.0002)	0.059
			Maximum likelihood	0.996 (0.992, 0.999)	0.017*
			*Q*-test		0.239
			MR-PRESSO (global test)		0.219

AD, Alzheimer’s disease; CRP, C-reactive protein; DLB, dementia with Lewy bodies; MR, Mendelian randomization; OR, odds ratio; PD, Parkinson’s disease. *p < 0.05.

## Discussion

In this study, we performed univariable MR and MVMR analyses to evaluate the association between the genetically determined fibrinogen and γ-fibrinogen levels on NDs. The results of our principal analyses did not find evidence that plasma fibrinogen and γ-fibrinogen levels were associated with AD, PD and LBD, with consistent results in MVMR analyses after adjusting for CRP and height. For AD, these findings are generally consistent with previous MR analyses showing no effect of genetically determined fibrinogen levels on AD (Fani et al., 2021). Our findings are less consistent with a previous reviews and meta-analysis that reported on significant associations between fibrinogen levels and AD. This inconsistency could be explained by the confounding factors and selection bias. Given that AD primarily affects the elderly, using an older age group could lead to selection bias due to recruitment on surviving exposure and competing risk (Schooling et al., 2019). To avoid selection bias, we used both proxy-case and clinically diagnosed AD GWAS in the current study. Moreover, we used the MVMR analysis to eliminate confounding factors. Additionally, using two-step MR analysis, we found that higher fibrinogen levels could contribute to the risk of AD via increasing CRP levels. A recent MR analysis using larger numbers of IVs indicated that genetically predicted high CRP levels might increase the risk of AD, which could support the positive association in the current study (Zhang et al., 2022). To our knowledge, there has been no MR study performed on the casual effect of fibrinogen on PD and LBD. A previous observational study found that inverse associations with PD were observed for fibrinogen levels among women (Ton et al., 2012). However, observational studies may be subject to confounding factors and reverse causation.

Inflammation is a common feature of NDs. Sustained inflammatory responses may contribute to neuronal damage and disease progression in NDs. Previous research has proposed possible mechanisms for the association between inflammation and NDs (Glass et al., 2010; Amor et al., 2014). Fibrinogen is involved in the pro-inflammatory process by binding to a number of immune cell sites (Davalos and Akassoglou, 2012). Inflammation could cause blood-brain barrier (BBB) damage and leakage during the pathogenesis of NDs. In human tissue and relevant animal models, fibrinogen is deposited in the central nervous system (CNS) of NDs via a leaky (Petersen et al., 2018). Deposited fibrinogen may disrupt the BBB, potentially contributing to CNS pathologies and promoting NDs neuropathology. In this study, we found that higher fibrinogen levels may associated with the risk of AD via increasing CRP levels. This association indicated that fibrinogen might be involved in the pathophysiology of AD as a trigger of the inflammatory system. However, fibrinogen and γ-fibrinogen were not associated with PD and LBD. We speculate that fibrinogen may be a product of the process by which inflammation causes NDs rather than the cause of these diseases. During the slow progression of NDs, long-term accumulation of fibrinogen produced in the inflammatory response may promote and aggravate the neurodegenerative.

Surprisingly, our findings provide suggestive evidence that genetically determined taller adult height is inversely associated with the risk of AD. This finding is consistent with a previous MR analysis (Larsson et al., 2017) and a case-control study (Petot et al., 2007) showing an inverse association between adult height and AD. Our study extends previous MR findings by using the largest GWAS of AD, including clinically diagnosed AD and AD-by-proxy individuals, and applying additional sensitivity analyses, including the Cochran *Q*-test, maximum likelihood estimate method and MR-PRESSO analysis. Previous studies speculated that childhood nutrition and height-related biological pathways could be associated with the risk of AD (Beeri et al., 2005; Wood et al., 2014). Although the underlying mechanisms of this association are unknown, we speculate that taller people secrete more growth hormone, which could stimulate cell division and growth, thus delaying the process of neurodegeneration. Moreover, recent studies suggest that adult height may be related to the risk of cancer (Choi et al., 2019), and cancer diagnosis has been proven to have an inverse effect on AD (Karanth et al., 2022). Its underlying pathway is still unclear. Further investigation may help explain the possible mechanism by which adult height is inversely associated with AD.

### Study Strengths

Our study had several strengths. First, the design of MR analysis minimizes residual confounding and avoids bias from reverse causation. Second, we calculated and selected SNPs with F statistics greater than 10 as valid instrumental variables to exclude the effect of weak instrument bias. Then, considering AD typically occurs in old age, participants need to survive AD exposure and competing risk to be recruited, resulting in a selection bias (Gkatzionis and Burgess, 2019). We used the largest GWAS data, including clinically diagnosed AD and AD-by-proxy participants, to minimize the impact of selection bias. Finally, we used the MVMR method to control for confounder bias from biomarkers and height in order to form robust and reliable conclusions. We also performed substantial sensitivity analyses to validate the MR model assumptions.

### Study Limitations

This study and MR analysis also had several limitations. First, only individuals of European descent were included which may restrict our ability to generalize the results. Second, genetic variables can affect an individual over the course of their lifetime. Thus, it is difficult to determine how short intervals of higher fibrinogen levels might influence the risk of developing AD, PD and LBD. Finally, pleiotropic effects amount the genetic instruments could result in deviated effect estimates; therefore, we performed a large quantity sensitivity analysis to minimize their impact.

In summary, the current MR study indicated that plasma fibrinogen levels might be associated with the risk of AD via increasing CRP levels. However, evidence from the current univariable MR and MVMR analysis did not support the presence of a causal association between plasma fibrinogen and γ-fibrinogen levels and PD and LBD. It suggests that fibrinogen may be involved in the inflammation pathway, which could relate to the pathogenesis of AD. Furthermore, our finding suggested that higher genetically determined height levels were inversely associated with an increased risk of AD. This signifies the possible role of height levels in the risk of developing AD under certain conditions. Further studies of underlying biological associations between fibrinogen and height with NDs would provide more insight into the etiology of NDs.

## Data Availability Statement

The original contributions presented in this study are included in the article/Supplementary Material, further inquiries can be directed to the corresponding author.

## Ethics Statement

Our analysis is a secondary analysis of published study or publicly available GWAS summary data. Ethics approval and consent statement for each GWAS in this study can be found in the original publications.

## Author Contributions

Both authors listed have made a substantial, direct, and intellectual contribution to the work, and approved it for publication.

## Conflict of Interest

The authors declare that the research was conducted in the absence of any commercial or financial relationships that could be construed as a potential conflict of interest.

## Publisher’s Note

All claims expressed in this article are solely those of the authors and do not necessarily represent those of their affiliated organizations, or those of the publisher, the editors and the reviewers. Any product that may be evaluated in this article, or claim that may be made by its manufacturer, is not guaranteed or endorsed by the publisher.

## Footnotes

1
http://www.r-project.org
